# Estimation of Phoneme-Specific HMM Topologies for the Automatic Recognition of Dysarthric Speech

**DOI:** 10.1155/2013/297860

**Published:** 2013-10-08

**Authors:** Santiago-Omar Caballero-Morales

**Affiliations:** Technological University of the Mixteca, Road to Acatlima K.m. 2.5, Huajuapan de León, 69000 Oaxaca, OAX, Mexico

## Abstract

Dysarthria is a frequently occurring motor speech disorder which can be caused by neurological trauma, cerebral palsy, or degenerative neurological diseases. Because dysarthria affects phonation, articulation, and prosody, spoken communication of dysarthric speakers gets seriously restricted, affecting their quality of life and confidence. Assistive technology has led to the development of speech applications to improve the spoken communication of dysarthric speakers. In this field, this paper presents an approach to improve the accuracy of HMM-based speech recognition systems. Because phonatory dysfunction is a main characteristic of dysarthric speech, the phonemes of a dysarthric speaker are affected at different levels. Thus, the approach consists in finding the most suitable type of HMM topology (Bakis, Ergodic) for each phoneme in the speaker's phonetic repertoire. The topology is further refined with a suitable number of states and Gaussian mixture components for acoustic modelling. This represents a difference when compared with studies where a single topology is assumed for all phonemes. Finding the suitable parameters (topology and mixtures components) is performed with a Genetic Algorithm (GA). Experiments with a well-known dysarthric speech database showed statistically significant improvements of the proposed approach when compared with the single topology approach, even for speakers with severe dysarthria.

## 1. Introduction

The term dysarthria was initially defined as “a collective name for a group of speech disorders resulting from disturbances in muscular control over the speech mechanism due to damage of the central or peripheral nervous system” [1, 2]. More recently, dysarthria is described as an impairment in one or more of the processes involved in speech production: respiration, phonation (control of the vocal folds for appropriate voice quality and valving of the airway), resonance (ability to alter the vocal tract, and resonating spaces for correct speech sounds), articulation, and prosody (appropriate emphasis and inflection) [3]. People affected by this condition often present significant deficiencies in oral communication and reduced intelligibility due to the characteristic paralysis, weakness, and incoordination of the speech musculature [4]. This affects all aspects in the life of dysarthric people, from social interaction to academic performance and vocational placement [5].

The damage of the nervous system that leads to dysarthria can be caused by congenital disorders (e.g., cerebral palsy), cerebrovascular accident (CVA), traumatic brain injury (TBI), or degenerative neurological disease such as Parkinson's or Alzheimer's disease. Some of these conditions as cerebral palsy and TBI produce nonprogressive dysarthria while Parkinson's and Alzheimer's disease produce a degenerative dysarthria that degrades speech over time [3].

**Figure 1:**
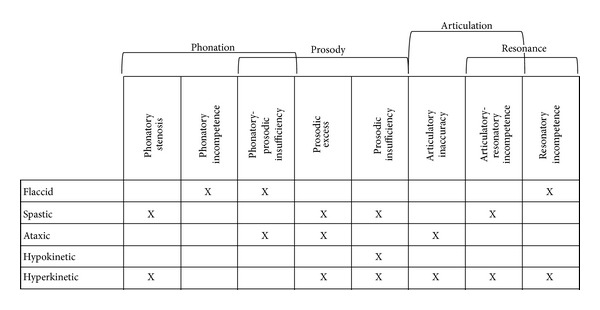
Speech impairments present in the different types of dysarthria.

The affected muscles in dysarthria may include the lungs, larynx, oropharynx and nasopharynx, soft palate, and articulators (lips, tongue, teeth, and jaw) [6]. The degree to which these muscle groups are compromised determines the particular pattern of speech impairment and the type (faccid, spastic, ataxic, hypokinetic, hyperkinetic) and level (mild, moderate, severe) of dysarthria. In Figure 1 a general overview of the speech impairments associated to each type of dysarthria as reported in [1] is presented, noting that most of the abnormalities are related to the processes of phonation, prosody, and articulation. Thus, dysarthric speech may include imprecise consonants and distorted vowels (segmental deficits), irregular articulatory breakdowns, excessive or equal stress to all syllables, and a slow rate of speech with a phonatory-prosodic insufficiency described as harsh, monotonous, and monoloudness [3]. Typical symptoms also include strained phonation, imprecise placement of the articulators, incomplete consonant closure resulting in sonorant implementation of many stops and fricatives, and reduced voice onset time (VOT) distinctions between voiced and unvoiced stops [4]. Because the range of speech impairments or abnormalities in dysarthric speech is extensive, assessment commonly requires different tests.

### 1.1. Effect of Dysarthria on Vowels, Consonants, and Prosody

Vowel distortion is common because dysarthric speakers produce individual movements or changes in overall vocal tract shape with reduced displacements and velocities [4]. This leads to the following specific abnormalities: large deviations and centralization in formant frequencies, change in vowel space area, shallower formant slopes, and greater interspeaker formant transition variability [4]. However, for dysarthric speakers, vowels are physically easy to produce since they do not require dynamic movement of the articulatory muscles.

In contrast to vowels, consonants require fine motor control of the articulators. Thus, these are more affected and difficult to produce in the presence of dysarthria [7]. This is particularly more evident for the consonants that involve some kind of frication event: a burst or transient noise (stops), a brief noise interval (affricates), or a longer noise interval (fricatives).

A metric used to measure the impairment of consonant production is the VOT, which is defined as the length of time that passes between the release of a stop consonant and the onset of voicing, the vibration of the vocal folds. Dysarthria is characterized by significant variability in VOT. For example, in [8] people with spastic dysarthria produced consonants with shorter VOT when compared with people with normal speech. In contrast, in [9, 10] long VOT durations were observed in people with spastic dysarthria. In [11] high VOT variability was observed among people with ataxic dysarthria.

About prosody, decreased range of pitch and loudness have been noted as prosodic characteristics of dysarthria [1] where structures of the language such as stress, rhythm, and intonation are affected [12]. Acoustic features associated with prosody include fundamental frequency, amplitude, duration, and segment quality [13], which are affected by the diverse impairments of speech musculature caused by dysarthria. This is important for communication because deficiencies in prosodic features interfere with the intelligibility of vowels, especially when tones are involved.

### 1.2. Phonatory Dysfunction

The speech impairments and abnormalities caused by dysarthria (see Figure 1) lead to phonatory dysfunction, which is one of the most frequently observed abnormalities perceived across all types of dysarthria [1, 2, 14–16]. Phonatory dysfunction is a primary feature noted in clinical assessment of dysarthria [16]. Because a phoneme is generally regarded as an abstraction of a set of speech sounds (phones) which are perceived as equivalent to each other in a given language, phonatory dysfunction may be linked more generally with communication deficits [16].

Slow rate of speech and timing produce prolonged phonemes, and this can make a single-syllable word to be perceived (or recognized) as a two-syllable word (*day*→*dial*). Also, words with long voiceless stops can be interpreted as two words because of the long silent occlusion phase in the middle of the target word (*before*→*be for*) [17, 18].

In automatic speech recognition (ASR) and perception tests, phonatory dysfunction leads to an increase of deletion, insertion, and substitution of phonemes [7, 17, 19, 20]. These characteristics decrease the speaker's intelligibility and restrict the speaker's phonetic repertoire, causing that some sounds or phonemes cannot be uttered or articulated correctly.

### 1.3. Speech Recognition Technology

People with dysarthria also have muscular atrophy, which restricts their ability to use switches or keyboards for communication or control of assistive devices (e.g., an electric wheelchair). In this case, speech recognition technology is an attractive alternative for communication and control despite the difficulty of achieving robust recognition performance because of phonatory dysfunction.

Many assistive applications based on speech recognition have been developed and implemented. In [17, 21–23] the use of commercial ASR systems for dictation (e.g., Dragon Naturally Speaking) was explored to improve speech communication. These studies showed varying levels of recognition performance in the range of 50% to 95% for users with different levels of dysarthria and small vocabularies (<50 words).

In contrast, in [19, 24–28] specific ASR systems were developed for dysarthric speakers. In [24] an ASR system for dysarthric speakers was built with Artificial Neural Networks (ANNs). In comparison with a commercial ASR, the ANN-based system achieved higher recognition performance. In [25, 26] ASR systems built with Hidden Markov Models (HMMs) [29] achieved significant performances for Dutch and Japanese dysarthric speakers, respectively. In [27] a HMM-based ASR system was able to achieve recognition accuracies over 80% for British speakers with severe dysarthria and a restricted vocabulary (7–10 words) to control electronic devices (e.g., radio, TV). In [30], a hybrid approach that integrated HMMs and ANNs was presented to improve recognition of disordered speech. In [19], a HMM-based ASR system for dysarthric speakers was built to control a web browser with 47 pre-selected words achieving accuracies within the range of 34.3% to 83.3%. In [31], a HMM-based ASR was integrated with speech synthesis to improve intelligibility of dysarthric speech. Recognition accuracies of 65% to 80% were achieved with improvements on intelligibility as measured by the Mean Opinion Score (MOS). Finally, in [28, 32], the development of a HMM-based voice-input voice-output (VIVOCA) communication aid device for people with disordered speech was presented. Such device was intended to recognize and interpret a dysarthric person's speech and generate a more intelligible (and clear) version of the same speech. This system achieved a mean accuracy of 67% in real usage situations with small vocabularies (14–47 words).

In general, ASR technologies are focused to identify (recognize) more accurately the sentences spoken by the dysarthric speaker independently of the severity of the dysarthria. This is very important for the development of applications (as those described above) which have the objective of improving communication and interaction with other people or other assistive systems.

### 1.4. Justification and Proposal

For people with dysarthric speech, the development of assistive technology based on ASR is dependent on the achievement of robust recognition performance. This is not an easy task because of the wide range of abnormalities associated to dysarthric speech. In addition, although high recognition performance is achieved (e.g., >80%), this is obtained after several speaker-specific training session. The associated costs, in money and time, cannot be afforded for most of the people with this disorder. Overcoming these difficulties is worthy as human-computer interaction using ASR is more suitable when the person with dysarthria also has muscular atrophy, which restricts the ability to use switches or keyboards. In such case, ASR interaction has been reported to be more efficient and faster than using mechanisms [23, 33–35].

It is proposed that an ASR system that can learn the speech patterns with the less amount of training data is suitable for dysarthric speakers. A previous approach considered the response of the ASR system to estimate the phoneme confusion patterns of the speaker's speech. Then, this information was integrated into the ASR process to correct those confusion errors (deletion, substitution, and/or insertion of phonemes) and provide a more accurate response [18, 26, 36–38]. This approach performed better than other approaches that made use of speaker adaptation techniques (as those used by commercial ASR systems) because, as commented in [39], these are insufficient to deal with the abnormalities present in dysarthric speech. However, the performance of the confusion-matrix approach depends on the output of the ASR system and thus of its performance.

HMMs were considered for this work because these are the most frequent techniques used for recognition of normal and disordered speech. This is due to the efficiency of the HMMs to model the variation in the statistical properties of speech, both in the time and the frequency domains [40].

In this work, an approach based on finding suitable phoneme-specific HMM parameters for the ASR system is presented. It is argued that this approach can improve the acoustic modelling of phonemes affected by dysarthria and improve the performance of ASR, and thus, of other post-processing techniques as the phoneme confusion-matrix approach.

Based on the characteristics of dysarthric speech which were presented in this section the following parameters were considered for optimization of the ASR process.

- *Topology*. This was considered an important parameter because in the works reviewed in Section 1.3 it was noted that for all phonemes or words in the ASR's vocabulary the same HMM topology was used. As discussed in Sections 1.1 and 1.2, there is a significant variability in the phonetic dysfunction of vowels and consonants. Hence, specific topologies should be used for the acoustic modelling of each phoneme in the dysarthric speaker's phonetic repertoire because not all phonemes are affected in the same way by dysarthria. For this work, the architecture (Bakis, Ergodic) and the number of states of the HMM were considered within the topology parameter. This is an extension on the work presented in [42], where topology optimization consisted in finding the optimal number of states for word HMMs considering just a Bakis left-to-right topology.
- *Number of Gaussian Mixture Components*. An important element of each state in a HMM are the emission probabilities. These represent the probabilities of an observation vector (e.g., the speech signal) being generated from a particular HMM state [43]. These probabilities are modelled by probability density functions (PDFs) which are represented as a weighted sum of Gaussians PDFs, each with different mean and variance [43, 44]. This is known as a Gaussian mixture, and the number of Gaussian PDFs affects the response of a HMM-based ASR system [4, 43, 45].

The selection of the suitable topologies and number of Gaussian mixture components for each phoneme in the dysarthric speaker's language was performed with a Genetic Algorithm (GA) which is an important tool used in the field of optimization [46]. The performance of the ASR with the resulting GA-HMMs were compared with the approach of developing a speaker-dependent (SD) system, where training of HMMs is performed with the speech data of the target speaker [17, 27, 32, 35, 39]. The proposed approach achieved statistically significant gains on ASR accuracy when tested with the SD approach on a well-known database of dysarthric speech (Nemours [41]).

The details of the proposed phoneme-specific approach are presented in this paper as follows. In Section 2, the details about the selection of the HMM topologies and number of Gaussian mixture components for each phoneme are presented. Then, in Section 3, the information regarding the structure and elements of the GA used for the optimization of the phoneme's HMM parameters is presented. In Section 4, the results obtained with the proposed approach and the comparison with the SD approach are presented. Finally, in Section 5 the results obtained are discussed and future work is presented.

## 2. HMM Parameters for Optimization

### 2.1. HMM Topology

**Figure 2:**
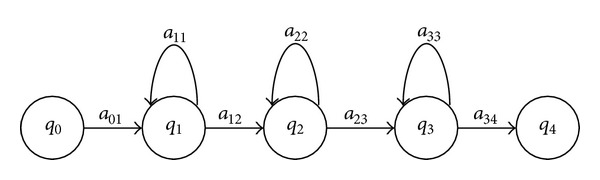
Three-state left-to-right Bakis topology of a HMM for modelling of phonemes (Bakis-1).

**Figure 3:**
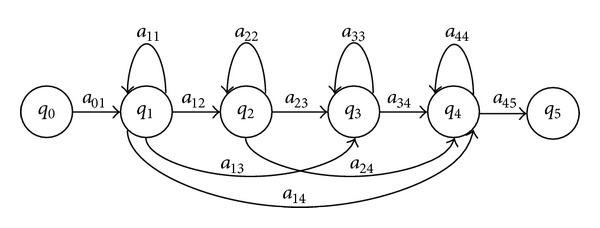
Four-state left-to-right Bakis topology of a HMM for modelling of phonemes with additional transitions (Bakis-2).

An important element of the HMMs is the topology or structure. In Figures 2 and 3 the topologies of HMMs for recognition of phonemes are shown. These topologies are known as Bakis, and the most frequently used is the three-state left-to-right structure [29, 31, 41, 43, 45] of Figure 2. Nowadays, commercial ASR system are based on phoneme HMMs with Bakis structure.

**Figure 4:**
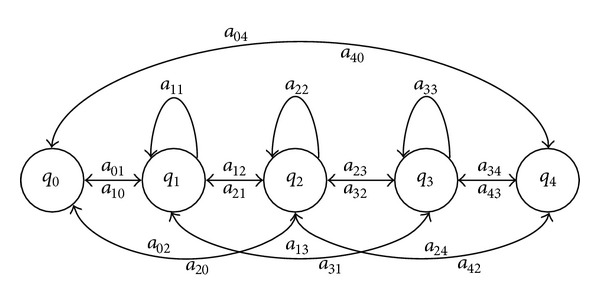
Ergodic structure of a HMM for modelling of words.

Another topology is known as Ergodic which is shown in Figure 4. In comparison with the Bakis topology, in the Ergodic topology every state (*q*) can be reached from every other state in a finite number of steps [29]. For recognition of dysarthric speech, Ergodic is commonly used when the ASR system is based on whole-word recognition [7, 27, 47].

In the works reviewed in Sections 1.3 and 1.4, the structure of the HMMs is fixed for the modelling of all phonemes or words in the vocabulary of the speech application. In this work it is argued that for dysarthric speech the topology must be specific for each phoneme because of the effect of phonatory dysfunction and the wide range of abnormalities caused by the affected speech articulator. Hence, slow pronunciation that affects speaking rate and timing may be more evident for certain phonemes than for others. In this case, an Ergodic topology could be more suitable to model speech with inconsistencies in speaking rate instead of a Bakis topology.

On the other hand, a Bakis structure with a large number of states has been reported to be suitable for modelling of long and poorly differentiated phonetic units [41]. Because of this, the number of states is an important element to be considered in the topology of HMMs for recognition of dysarthric speech. The number varies from the standard three states [35, 45] to eight [41] and 11 states [32]. Thus, for the optimization of the topology the following levels were considered for each parameter:

- type: Bakis of Figure 2 (Bakis-1), Bakis of Figure 3 (Bakis-2), and Ergodic of Figure 4 (three levels);
- number of states for each type: 3-to-11 (nine levels).

### 2.2. Gaussian Mixture Components

Another parameter considered for optimization is the number of Gaussian mixture components used for each HMM state. This parameter is important for the modelling of the emission probabilities, which represent the probabilities of an observation vector (e.g., the speech signal) being generated from a particular HMM state [43]. These probabilities are modelled as a weighted sum (mixture) of Gaussians PDFs, each with different mean and variance [43, 44].

The number of Gaussian PDFs (mixture components) affects the performance of a HMM-based ASR system [4, 43, 45]. High ASR performance is obtained with a number of Gaussian mixture components within the range of eight [38] to 16 [45]. However, usually less than ten Gaussian mixture components are used [35]. Thus, for the optimization of the number of mixture components 16 levels were considered based on these ranges 1-to-16.

In the following section, the details of the optimization method used for these parameters is presented.

## 3. Optimization Method: Genetic Algorithm

The selection of HMM topologies and number of mixture components is performed by a micro-Genetic Algorithm (micro-GA), which is a computational method based on Darwin's rules of natural selection. A GA is a search heuristic that mimics the process of natural evolution and generates useful solutions for optimization problems [46].

**Figure 5:**
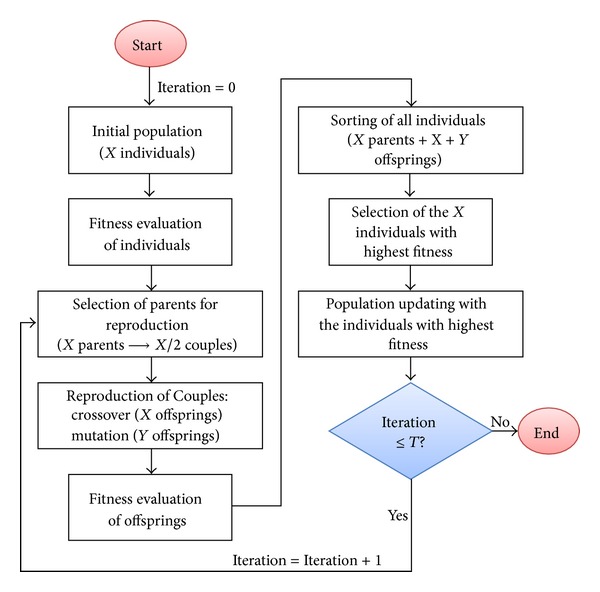
Structure of the micro-GA.

In general, GAs have been used in the field of ASR research for the optimization of HMMs as presented in [42, 48, 49]. In [42, 49], a GA was used to optimize the observation probabilities and transition states for HMM-based ASR systems. In contrast with the proposed approach, in [42], the topology optimization consisted in finding the optimal number of states for word HMMs considering just the Bakis-1 left-to-right topology. The proposed approach extends on the work presented in [42] by eliminating the left-to-right restriction and considering other topologies as the Bakis-2 and Ergodic with more states. In Figure 5, the general structure of the micro-GA used for this work is presented.

The GA starts with an “Initial Population” of candidate solutions or “Individuals”. Each of these solutions is evaluated to assess its “Fitness” which is related to the problem to be solved. In this case, the problem consists in finding the assignation of topologies and number of mixture components that would increase ASR performance, and each individual represents a set of assignations. Then, fitness is evaluated as the recognition accuracy obtained with the assignations given by an individual.

These individuals are selected for “Reproduction” based on Darwin's rule of “survival of the fittest” (e.g., the individuals with better fitness). It is expected that, as happens in nature, the individuals with better characteristics survive, reproduce, and produce “Offsprings” which inherit the characteristics of their “Parents” which are refined after some generations. Then, for this case, the individuals of the initial population become the parents for new solutions (offsprings) which are constructed by reproduction operators. It is expected that good solutions (assignation of topologies and number of mixture components) will produce better solutions. The selection of parents is performed based on their fitness.

After reproduction, the offsprings are evaluated to assess their fitness. If the offsprings are better than other individuals in the population then these will be replaced by them. The process iterates until no change in the overall fitness of the entire population is achieved (or after a given number of iterations).

In comparison with a conventional GA, a micro-GA can work with a very small initial population (typically four or five individuals [50]) which can be randomly generated. This algorithm can converge (e.g., to find an optimal solution) quickly within a few iterations and provide estimates as good as a conventional GA, where populations can be up to 1000 individuals. In the following sections the details of the micro-GA are presented.

### 3.1. Initial Population

The micro-GA starts with 10 individuals where

- the 1st individual consists in the assignation of the Bakis topology of Figure 2 with three-states (Bakis-1) for all phonemes;
- the 2nd individual consists in the assignation of the Bakis topology of Figure 3 with four-states (Bakis-2) for all phonemes;
- the 3rd individual consists in the assignation of the Ergodic topology of Figure 4 with three-states for all phonemes;
- the 4th-to-10th individuals are randomly generated assignations of the Bakis and Ergodic topologies mentioned above with a number of states within the nine levels specified in Section 2.1.

**Figure 6:**
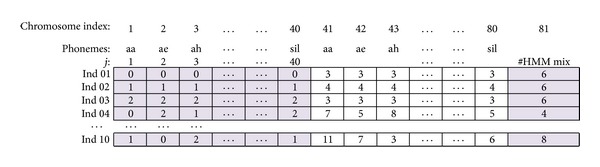
Chormosome representation of the topology and number of mixture Gaussian components assignments.

In order to perform the reproduction of these individuals, the assignations are coded into “Chromosomes”, which are presented in Figure 6. Each solution is represented by a vector with 81 “Genes” or values where

- from gene *i* = 1,…, 40 the numbers represent the topology to be assigned to the *j*th phoneme (in this case, *i* = *j*): Bakis-1 (0), Bakis-2 (1), Ergodic (2);
- from gene *i* = 41,…, 80 the numbers represent the number of HMM states considered for the topology assigned to the *j*th phoneme (3–11);
- gene 81 represents the number of Gaussian mixture components used for acoustic modelling with the HMMs (1–16).

### 3.2. Fitness Evaluation

For each individual in the population, a set of HMMs is built with the assigned topologies. The parameters of these HMMs as emission and transition probabilities are estimated with the Baum-Welch and Viterbi algorithms [43]. This process, called supervised training, is performed with a set of “training” speech.

Then, to measure the “Fitness” of each individual, the % Word Recognition Accuracy (WAcc) is computed on a set of “testing” speech. This measure is computed as

(1) $$\begin{array}{c} \text{WAcc}=\frac{N-D-S-I}{N}, \end{array}$$

where *N* is the number of elements (words or phonemes) in the correct transcription of the spoken speech, and *D*, *S* and *I* are the number of elements deleted, substituted, and inserted in the output generated by the HMM-based ASR system when compared to the correct transcription.

### 3.3. Selection of Parents

For this process, the Roulette Wheel selection [51] was performed as follows.

(1) For each *x* = 1,…, *X* individual in the population its fitness *F*_*x*_ is computed as specified in Section 3.2.
(2) Compute the selection probability for each *x* individual as
  (2) $$\begin{array}{c} S\left(x\right)=\frac{F_{x}}{\sum_{x=1}^{X}F_{x}}. \end{array}$$
 If there is a situation where negative *F*_*x*_ values are obtained, the most negative *F*_*x*_ is taken as reference. Then, the absolute value of the reference is added to all *F*_*x*_ values in the population. In this way, the most negative value gets a fitness of 0, and the individuals with less negative *F*_*x*_ get new positive (but small) values. The individuals with positive *F*_*x*_ get their fitness increased accordingly to the absolute of the reference value. This adjustment does not change the concept of fitness as a value of 0 represents an individual with very poor abilities to solve the problem.
(3) Compute the cumulative probability *C*(*x*) for each individual as *C*(*x*) = ∑_*j*=1_^*x*^ *S*(*x*).
(4) Generate a uniform random number *r* ∈ {0,1}.
(5) If *r* < *C*(*x*), then select the first individual (*x* = 1), otherwise, select *x* such that *C*(*x* − 1) < *r* ≤ *C*(*x*).
(6) Repeat Steps 4 and 5 *X* times until all *X* individuals are selected.

This procedure gives as output *X*/2 pairs or couples of parents which then can produce offsprings by means of the reproduction operators known as crossover and mutation.

### 3.4. Reproduction of Parents

The reproduction operators enable the creation of offsprings (new solutions) from an initial set of individuals (parents). This process is equivalent to exploring points within the solution space of a problem: parents are initial solutions for the problem, and creating offsprings is equivalent to finding other solutions for the same problem.

The first reproduction operator is known as “Crossover” and consists in the interchange of genes between the parent's chromosomes [46]. Crossover is explorative as it makes a jump to a region somewhere “in between” two (parents) regions [52]. Because of this, the crossover operator diversifies the population [53].

There are many crossover schemes, and the use of a particular scheme depends of the kind of choromosome codification and type of problem to be solved. In this case, the chromosome represents assignations that represent topologies and number of Gaussian mixture components, and the values of each gene are positive integer numbers. For this codification, the linear crossover was used [54–56]. Considering two parent choromosomes, *A* = {*a*_1_,…, *a*_*m*_} and *B* = {*b*_1_,…, *b*_*m*_}, where *a*_*i*_ and *b*_*i*_ are the *i*th genes and *m* is the length of the chromosome, the genes for the offsprings *C* and *D* are obtained as

(3) $$\begin{array}{c} c_{i}=\alpha \times a_{i}+\left(1-\alpha \right)\times b_{i},\\ d_{i}=\alpha \times b_{i}+\left(1-\alpha \right)\times a_{i}, \end{array}$$

where *α* is a weight value which in this case was associated to the crossover probability. In this way, each *i*th gene of an offspring is a new value created from the arithmetic combination of genes at the same *i*th position of the parent chromosomes [56]. Note that, from the selection method presented in Section 3.3, *X* parents form *X*/2 couples, and each couple produces two offsprings; thus, *X* offsprings are created with the crossover operator.

The second reproduction operator is known as “Mutation” and consists in changing (randomly or based on a probability) a number of genes across all individuals. Thus, the mutation operator can create new individuals by making changes in a single individual. Mutation is exploitative as it creates random small deviations, thereby staying near (in the region of) the parent [52]. While the crossover operator diversifies the population, the mutation operator exploits the new result [53].

The changes performed by the mutation operator may consist in just selecting a gene and assigning it a different value within the associated allowable range, or selecting two genes and interchange their values. For this case, a number of *Y* parents are randomly selected from the initial population. Then, for each selected parent, the values of *h* randomly selected genes are changed with values within the associated ranges. The *Y* and *h* numbers are associated with the mutation probability *β* as follows:

(4) $$\begin{array}{c} Y=\text{round}\;\left(\beta \times X\right),\\ h=\text{round}\;\left(\beta \times m\right). \end{array}$$

This leads to *Y* changed parents that become the offsprings generated by mutation. In this work, the probabilities of both operators are related based on the following equivalence:

(5) $$\begin{array}{c} \alpha =1-\beta . \end{array}$$

**Figure 7:**
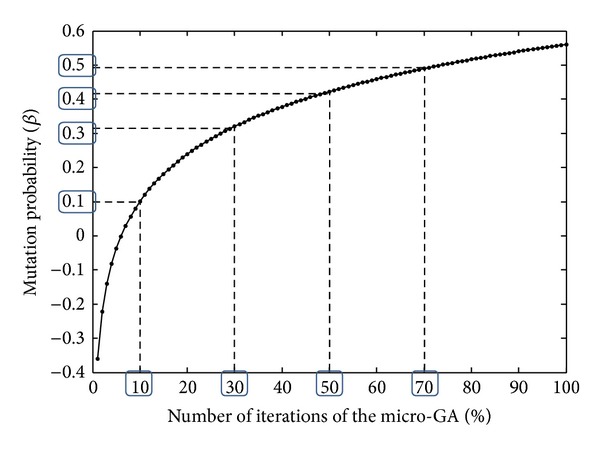
Logarithmic plot used for the estimation of *β*.

The probability of mutation was set as the reference for the crossover probability. The probability of mutation was considered to be increased depending on the number of iterations or generations of the GA. This increment was considered according to the expression *β* = (1/5)∗log⁡⁡(*w*) − 0.36 which is plotted in Figure 7. For this expression, *w* represents the percentage of the number of iterations of the micro-GA.

From Figure 7, four values for *β* are considered: 0.10, 0.30, 0.40, and 0.50. If *T* is defined as the number of iterations of the micro-GA, these *β* values are considered when the algorithm reaches *w* = 10%, 30%, 50%, and 70% of *T*, respectively. This form of estimation for *β* was considered in order to dynamically change the intensity of the explorative and exploitative searching process performed with the reproduction operators.

Initially, at iteration = 0, *β* = 0, thus *α* = 1.0 and just crossover is performed for all *X* individuals in the initial population. When the GA reaches, 10% of the total number of iterations (*T*), *β* = 0.10 and *α* = 0.9, thus crossover is performed on *X* = 9 individuals and mutation is performed on *Y* = 1 individuals. This value for *β* is kept until the GA reaches 30% of *T*, where *β* = 0.30 and *α* = 0.70, leading to crossover being performed on *X* = 7 individuals and mutation being performed on *Y* = 3 individuals. Then, this crossover and mutation rate are kept until the GA reaches 50% of *T*, where *β* = 0.40 and *α* = 0.60. This continues until the GA reaches 70% of *T*, where *β* = *α* = 0.50.

In this way, at the beginning of the GA explorative searching is mainly performed, thus intensifying diversification. As the GA continues, the exploitative search is increased until both are performed with the same intensity. Finally, the levels considered for *α* and *β* are consistent with other GA implementations [53, 55].

### 3.5. Stop Condition

As commented in [52], there are many stop criteria for a GA, like considering a maximum number of generations or iterations, a maximum number of functional evaluations, or convergence is achieved. For the micro-GA, a fixed number of iterations was considered, in this case *T* = 30. This is consistent with the stop condition of the GA presented in [42] (stop after 30 iterations). In experiments, however, it was observed that changes in convergence were minimal after 20 iterations.

## 4. Experiments on Dysarthric Speech

### 4.1. Speech Data

For the experiments the Nemours database of dysarthric speech was used [41]. This database has been widely used in ASR research as presented in [31, 36, 45, 57, 58]. The Nemours database consists of speech data from ten American-English speakers with dysarthrias resulting from either cerebral palsy or head trauma with associated quadriplegia [41]. The main speech data consists of a collection of 74 short sentences spoken by each speaker (740 sentences in total). These sentences are nonsense phrases that have a simple syntax of the form “the *X* is *Y* the *Z*”, where *X* and *Z* are monosyllabic nouns (74 in total) and *Y* is a bisyllabic verb (37 in total) in present participle form. Specific sentences were generated by randomly selecting *X* and *Z* (*X* ≠ *Z*) without replacement from the set of 74 nouns, and selecting *Y* without replacement from the set of 37 verbs. This process produced the first 37 sentences, and the other 37 sentences were generated by swapping the *X* and *Z* words in the first set. Because of this, in the complete set of 74 sentences there are two pronunciations of each noun and verb. The vocabulary in this set consists of 111 different words.

With this speech data, an initial assessment and recognition test were performed with human listeners. Because this paper is focused on the recognition task, the recognition scores produced by the human listeners for each dysarthric speaker are presented in Table 1. This information is important to identify the speakers with severe, moderate and mild levels of dysarthria based on their recognition scores. More information about this test and the intelligibility assessment for these speakers can be found in [41, 59]. 

 Based on the data presented in Table 1 and the identification presented in [45], the speakers were classified as presented in Table 2. In [45], four speakers were considered as moderate and three as mild. In this work, a speaker from the moderate group was taken to the mild group according to the data presented in Table 1 [41]. This classification is important for the training scheme of the SD systems presented in Section 4.2.

 In addition, two readings of narratives identified as “My Grandfather” and “The Rainbow” are included per speaker. While the sets of 74 sentences are phonetically and orthographically labelled, the narratives are not labelled at any level. Thus, extra labelling was performed to consider this speech material. The narratives were separated into sentences, leading to 18 sentences for the “My Grandfather” narrative, and 14 sentences for the “The Rainbow” narrative. The vocabulary in these narratives consisted of 158 different words. In Table 3 the selection of sentences used for training, fitness evaluation, and testing of the GA-HMMs is presented. This selection was defined to include all phonemes present in the speech database in the training, fitness evaluation, and testing sets.

 All speech data was coded into MFCC format where the front-end used 12 MFCCs plus energy, delta and acceleration coefficients. Also a frame period of 10 msec with a Hamming window of 25 msec and 26 filter-bank channels were used [43].

### 4.2. ASR Systems

The implementation tool for the HMMs and the recognition tasks was performed with HTK [43]. Because in the Nemours database 40 phonemes were identified, 40 monophone acoustic HMMs were constructed for each type of ASR system. In this work, two ASR systems were considered.

**Figure 8:**
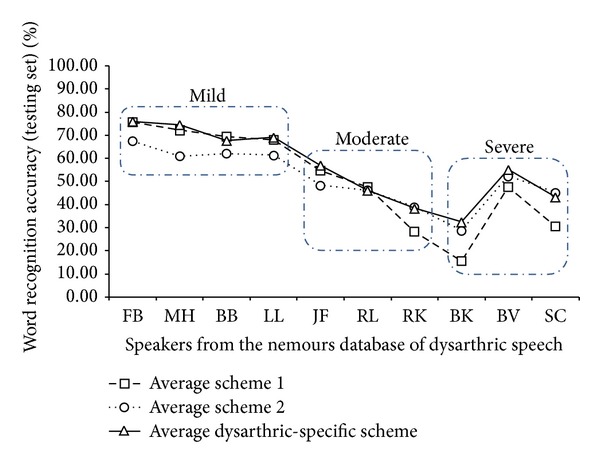
Average recognition accuracies obtained with different training schemes for the baseline SD ASR systems across all dysarthric speakers.

1. *Speaker-Dependent (SD) ASR*. The HMMs have the same parameters (topology, number of states, number of Gaussian mixture components per state) for all phonemes, and these are trained (built) with speech data of the target (test) speaker. This is the common approach for the development of ASR for dysarthric speech as performed in [17, 27, 32, 35, 39]. Thus, this ASR provides the baseline (or reference) performance for comparison purposes. About the parameters for this SD ASR, in Section 3.1 was determined that the first three individuals in the initial population for the micro-GA were ASR systems built with the topologies presented in Figures 2, 3, and 4, covering standard Bakis and Ergodic topologies. Hence, three baseline SD ASR systems were built as reference systems: SD Bakis-1, SD Bakis-2, and SD Ergodic.
2. Additionally, two schemes for building the SD ASR were considered: (1) using the training data from all speakers, including the target (test) speaker and (2) using the training data from just the target (test) speaker. Note that, if under the scheme (1) no speech data from the target speaker were used, then the system would be completely speaker-independent (SI), and thus, an adaptation technique would be required. In Table 4 the percentage of recognition accuracy obtained by the SD ASR systems under these two training schemes on the testing sets across all speakers is presented.
3. As presented, on average, speakers with mild to moderate dysarthria achieve higher recognition performance when training of the SD also includes speech data from other speakers. However, speakers with more severe dysarthria achieve better performance when the SD is trained only with speech data of the target speaker. This situation was also observed in [17, 45]. Because of these results, dysarthric-specific schemes were considered for training of the SD ASR systems:
  - mild scheme: training speech data from speakers FB, MH, BB, LL was used to train the SD for the same speakers;
  - moderate scheme: training speech data from speakers JF, RL, RK was used to train the SD for the same speakers;
  - severe scheme: training speech data from speakers BK, BV, SC was used to train the SD for the same speakers.
4. In Table 5, the recognition results obtained with the dysarthric-specific schemes are presented. In Figure 8, all schemes are presented for comparison. With the dysarthric-specific training schemes it was possible to achieve similar performance (and in some cases, higher performance) when compared with the schemes presented in Table 4. This is important because an ASR can be built for specific categories or levels of dysarthria. This may be the reason why, as observed in [17, 45], speakers with mild dysarthria get more benefits from using ASR built with normal speech (adapted speaker-independent ASR system) than by using SD ASR systems (in this case, mild dysarthric speakers are closer to normal speech than moderate or severe dysarthric speakers). Thus, for the optimization of the HMMs, the dysarthric-specific scheme was used for the creation of the baseline SD ASR systems.
5. *GA-Optimized (GA-op) SD ASR*. The HMMs of the baseline SD ASR are optimized with the micro-GA presented in Section 3 to make each HMM specific to the characteristics of the phoneme. Optimization involves the identification of the most suitable topology and number of Gaussian mixture components to improve the performance of the ASR system. For the micro-GA, training-independent speech data was used for fitness evaluation (see Table 3).

### 4.3. Convergence of the Micro-GA

**Figure 9:**
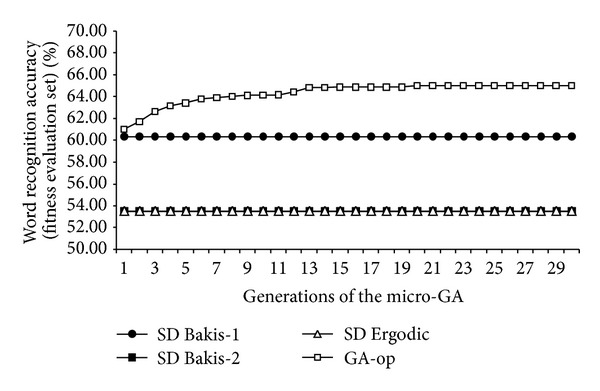
Convergence plot of the micro-GA.

In Figure 9, the average convergence plot of the GA-op SD HMMs across all iterations of the micro-GA on the fitness evaluation set is presented. This illustrates that the assignment of different topologies can lead to improvement on ASR performance. In Table 6, the assignations obtained with the micro-GA for each speaker's set of phonemes are presented.

### 4.4. Performance of the GA-op ASR System

**Figure 10:**
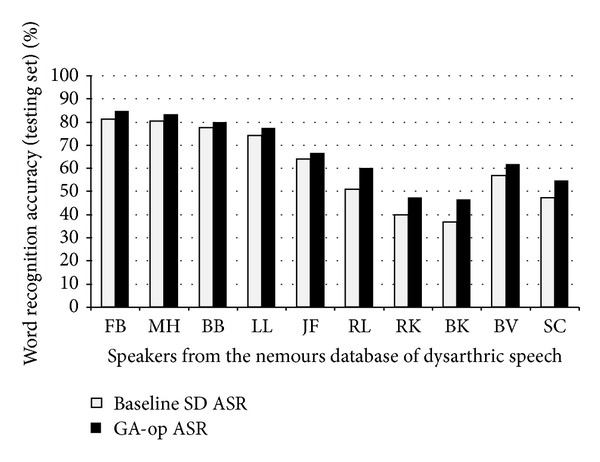
Recognition accuracies (WAcc) on the testing set with the baseline SD ASR and GA-op ASR systems.

In Table 7 and Figure 10 the comparison of performances of the baseline SD ASR systems with the GA-op SD ASR is presented. The GA-op was compared with the baseline SD ASR system with the higher performance from Table 5. As presented, the performance of the GA-op SD ASR system is higher than the baseline's for each speaker independently of the dysarthric level.

 On average, an increase of 5.3% was achieved across all dysarthric speakers (66.20%–60.90%). These results were statistically significant with a *P* value <0.10 using the matched-pairs test described in [60]. It is acknowledged that, in practical terms, ASR should be higher as is for normal speech (80%–96% for small vocabularies) [61]; however, human recognition for dysarthric speech has been reported to be accurate between 7% and 61% of the time [45, 62]. The performance achieved with the proposed approach is higher than human recognition based on this information. 

## 5. Discussion and Future Work

In this work an approach consisting of modelling each phoneme with a specific topology for dysarthric speech was presented. Initially the approach of developing SD ASR systems was considered. Results presented in Figure 8 showed that by adding speech samples from other speakers with similar level of dysarthria to the training of the SD ASR system, higher performance could be achieved for some speakers. As presented in Table 7 and Figure 10, when optimizing the topologies of the HMMs of this SD ASR system more gains in recognition performance were obtained. In practice, this approach can be applied if the speaker already has a SD ASR system, or for the designing of dysarthric-specific SD ASR systems. In such cases, an automatic trainer and builder of ASR systems must be developed. In the field of human-computer interaction (HCI), this approach can be used to further improve the efficiency of assistive interfaces.

From Table 6 it is observed that the Bakis-1 topology is the most suitable for all speakers with mild dysarthria, and just for some with moderate and severe dysarthria. In Particular for speaker RL the topologies Bakis-1, Bakis-2, and Ergodic were equally assigned to different phonemes with an average of seven states and 12 Gaussian mixture components per state. This speaker was classified with moderate dysarthria based on the recognition results presented in Table 2. However the assignations estimated by the micro-GA indicate that this speaker may present a wide range of variations in the pronunciation of phonemes (and, thus, more specific HMMs must be considered). Note than an improvement of almost 10% was obtained for speaker RL after the optimization of the HMMs of the baseline SD ASR system.

For speakers BK and BV, which were classified with severe dysarthria, the most suitable topologies were Bakis-1 and Bakis-2 (with some Ergodic), respectively. However note that the average number of states is significantly higher (seven and eight resp.) than for the mild and moderate speakers (excluding speaker RL). For speaker BV, the number of Gaussian mixture components is the highest with 16.

In general, speakers RL, BK, and BV required more states for the acoustic modelling of their phonemes. Speakers FB, MH, BB, LL (mild dysarthria), and JF (moderate dysarthria) required less states, with an average of four-to-six states. Speaker RK, which was classified with moderate (but close to severe) dysarthria, required less states with an average of four. Speaker SC, which is the one with the lowest recognition performance in Table 2 and was classified with severe dysarthria, required an average of six states.

Although for speakers FB, MH, BB, LL, JF, RL, BK, and BV clear assignations were observed based on their level of dysarthria (e.g., mild to severe dysarthria leads to increase the number of HMM states for acoustic modelling), for speakers RK and SC these were not observed (e.g., assignations for the moderate-to-severe and severe RK and SC speakers are more associated to a mild dysarthric speaker). However, the improvements achieved with the proposed approach was consistent across all speakers and levels of dysarthria.

Overall, the standard three-state left-to-right Bakis topology used for acoustic modelling of phonemes requires more states for the modelling of dysarthric speech. And in some cases, more than one type of topology is required. Also, this is dependent of the acoustic characteristics of each phoneme in the speaker's repertoire.

Future work is focused on extending the study of the situations observed in this work:

- to explore on the use of dynamic topologies, where besides changing the number of states, the transitions between them can also be changed;
- to improve the convergence of the micro-GA with alternative crossover and mutation operators;
- to test the approach on a different and larger database of dysarthric speech (e.g., the TORGO database);
- to incorporate the post-processing confusion-matrix approach presented in [18, 26, 38] for further improvement (for this, more dysarthric speech data would be required);
- to explore on the use of the HMM assignations for the assessment of dysarthric speech.

## Figures and Tables

**Table 1:** Average percentage of correct identifications for the speakers of the nemours database (taken from [41]).

Talker	Average	Talker	Average
BB	89.7	LL	84.4
BK	58.2	MH	92.1
BV	57.5	RK	68.6
FB	92.9	RL	73.3
JF	78.5	SC	51.5

**Table 2:** Classification of dysarthric speakers according to assessment tests.

Speaker	Level of dysarthria	Speaker	Level of dysarthria
FB	Mild	RL	Moderate
MH	Mild	RK	Moderate
BB	Mild	BK	Severe
LL	Mild	BV	Severe
JF	Moderate	SC	Severe

**Table 3:** Classification of speech data for training, fitness evaluation, and testing tasks.

	Grandfather			Rainbow		Nonsense						
Training	1	7	13	1	11	1	5	9	23	27	41	73
	2	8	14	2	12	2	6	10	24	28	42	74
	3	9	15	3		3	7	21	25	29	43	
	4			4		4	8	22	26	30	44	

Fitness evaluation	5	10		5	13	11	15	19	33	37		
	6	11		6	14	12	16	20	34	38		
						13	17	31	35	39		
						14	18	32	36	40		

Testing	12			7		45	49	53	57	61	65	69
	16			8		46	50	54	58	62	66	70
	17			9		47	51	55	59	63	67	71
	18			10		48	52	56	60	64	68	72

**Table 4:** Recognition accuracies obtained with different training schemes for the baseline SD ASR systems across all dysarthric speakers.

Speaker	Training scheme 1			
	SD Bakis-1	SD Bakis-2	SD Ergodic	Average scheme 1
FB	77.73	72.93	76.42	75.69
MH	73.80	69.43	73.36	72.20
BB	75.69	63.30	69.27	69.42
LL	69.20	67.86	66.96	68.01
JF	60.89	48.00	55.56	54.82
RL	50.67	48.44	43.56	47.56
RK	44.30	15.35	25.44	28.36
BK	13.27	6.64	26.99	15.63
BV	48.64	50.91	43.64	47.73
SC	44.55	19.55	27.73	30.61

Speaker	Training scheme 2			
	SD Bakis-1	SD Bakis-2	SD Ergodic	Average scheme 2

FB	72.49	63.76	65.94	67.40
MH	66.38	58.95	57.64	60.99
BB	69.72	54.13	62.39	62.08
LL	64.73	57.14	62.05	61.31
JF	49.78	43.11	52.00	48.30
RL	51.11	42.67	44.89	46.22
RK	38.16	39.04	39.47	38.89
BK	31.42	18.58	36.28	28.76
BV	55.91	53.18	47.73	52.27
SC	44.09	43.18	48.18	45.15

**Table 5:** Recognition accuracies obtained with dysarthric-specific training schemes for the baseline SD ASR systems across all dysarthric speakers.

Speaker	SD Bakis-1	SD Bakis-2	SD Ergodic	Average
Mild scheme				
FB	81.22	69.43	76.86	75.84
MH	80.35	72.49	70.31	74.38
BB	77.52	61.47	63.76	67.58
LL	74.11	66.96	65.63	68.90

Moderate scheme				
JF	64.00	54.67	52.00	56.89
RL	51.11	47.11	40.00	46.07
RK	39.47	35.53	39.91	38.30

Severe scheme				
BK	36.73	30.09	30.53	32.45
BV	56.82	53.64	54.55	55.00
SC	47.27	40.45	41.82	43.18

**Table 6:** HMM assignations obtained by the micro-GA for the phonemes of each dysarthric speaker.

	Phonemes	FB		MH		BB		LL		JF		RL		RK		BK		BV		SC	
		Type	States	Type	States	Type	States	Type	States	Type	States	Type	States	Type	States	Type	States	Type	States	Type	States
1	“aa”	Bakis-1	5	Bakis-1	3	Bakis-1	5	Bakis-1	5	Bakis-1	5	Bakis-2	11	Bakis-1	3	Bakis-1	7	Bakis-2	10	Bakis-1	5
2	“ae”	Bakis-1	5	Bakis-1	3	Bakis-1	4	Bakis-1	4	Bakis-1	4	Ergodic	7	Bakis-1	3	Bakis-1	6	Bakis-2	10	Bakis-1	6
3	“ah”	Bakis-1	7	Bakis-1	3	Bakis-1	3	Bakis-1	4	Bakis-1	4	Ergodic	8	Bakis-2	5	Bakis-1	5	Ergodic	7	Bakis-1	5
4	“ao”	Ergodic	8	Bakis-1	3	Bakis-1	5	Bakis-1	5	Bakis-1	4	Bakis-1	6	Bakis-1	3	Bakis-1	7	Bakis-2	10	Bakis-1	6
5	“aw”	Bakis-1	3	Bakis-1	3	Bakis-1	4	Bakis-1	4	Bakis-1	3	Bakis-2	8	Bakis-1	3	Bakis-1	7	Bakis-2	5	Bakis-1	5
6	“ay”	Bakis-1	3	Bakis-1	3	Bakis-1	5	Bakis-1	4	Bakis-1	3	Bakis-1	5	Bakis-1	3	Bakis-1	5	Bakis-2	10	Bakis-1	6
7	“eh”	Bakis-1	5	Bakis-1	3	Bakis-1	4	Bakis-1	4	Bakis-1	5	Bakis-1	7	Bakis-1	3	Bakis-1	5	Bakis-2	4	Bakis-1	6
8	“er”	Bakis-1	7	Bakis-1	3	Bakis-1	3	Bakis-1	5	Bakis-1	5	Ergodic	10	Bakis-1	3	Bakis-1	5	Bakis-2	3	Bakis-1	5
9	“ey”	Bakis-1	7	Bakis-1	3	Bakis-1	5	Bakis-1	4	Bakis-1	4	Bakis-1	4	Bakis-1	3	Bakis-1	7	Bakis-1	7	Bakis-1	7
10	“ih”	Bakis-1	7	Bakis-1	3	Bakis-1	5	Bakis-1	4	Bakis-1	3	Ergodic	8	Bakis-1	3	Bakis-1	7	Bakis-2	10	Bakis-1	5
11	“iy”	Bakis-1	5	Bakis-1	6	Bakis-1	6	Bakis-1	5	Bakis-1	4	Bakis-2	7	Bakis-1	3	Bakis-1	6	Bakis-2	10	Bakis-1	6
12	“ow”	Bakis-1	5	Bakis-1	3	Bakis-1	5	Bakis-1	5	Bakis-1	4	Bakis-2	6	Bakis-1	3	Bakis-1	6	Bakis-2	10	Bakis-1	4
13	“oy”	Bakis-1	5	Bakis-1	3	Bakis-1	3	Bakis-1	5	Bakis-1	5	Bakis-2	9	Bakis-1	3	Bakis-1	5	Bakis-2	11	Bakis-1	5
14	“uh”	Bakis-1	3	Bakis-1	3	Bakis-1	4	Bakis-1	5	Bakis-1	3	Bakis-2	11	Bakis-1	3	Bakis-1	7	Bakis-2	10	Bakis-1	6
15	“uw”	Bakis-2	10	Bakis-1	3	Bakis-1	8	Bakis-1	5	Bakis-1	3	Bakis-1	9	Bakis-1	3	Bakis-1	7	Ergodic	10	Bakis-1	7
16	“b”	Bakis-1	5	Bakis-1	3	Bakis-1	4	Bakis-1	4	Bakis-1	4	Bakis-1	4	Bakis-1	3	Bakis-1	7	Bakis-2	10	Bakis-1	4
17	“ch”	Bakis-1	7	Bakis-1	6	Bakis-1	5	Bakis-1	4	Bakis-1	5	Bakis-2	6	Bakis-1	3	Bakis-1	6	Ergodic	5	Bakis-1	7
18	“d”	Bakis-1	4	Bakis-1	3	Bakis-1	3	Bakis-1	4	Bakis-1	5	Ergodic	11	Bakis-1	3	Bakis-1	6	Bakis-2	9	Bakis-1	3
19	“dh”	Bakis-1	5	Bakis-1	3	Bakis-1	6	Bakis-1	4	Bakis-1	5	Ergodic	10	Bakis-1	3	Bakis-1	6	Bakis-2	10	Bakis-1	6
20	“f”	Bakis-1	3	Bakis-1	3	Bakis-1	5	Bakis-1	5	Bakis-1	4	Ergodic	8	Bakis-1	3	Bakis-1	7	Bakis-2	3	Bakis-1	6
21	“g”	Bakis-1	5	Bakis-1	3	Bakis-1	4	Bakis-1	4	Bakis-1	4	Ergodic	4	Bakis-1	3	Bakis-1	5	Bakis-2	3	Bakis-1	4
22	“hh”	Bakis-1	5	Bakis-1	3	Bakis-1	4	Bakis-1	4	Bakis-1	4	Ergodic	10	Bakis-1	3	Bakis-1	5	Bakis-2	10	Bakis-1	5
23	“jh”	Bakis-1	7	Bakis-1	3	Bakis-1	4	Bakis-1	5	Bakis-1	5	Ergodic	9	Bakis-1	3	Bakis-1	5	Bakis-2	6	Bakis-1	6
24	“k”	Bakis-1	5	Bakis-1	3	Bakis-1	5	Bakis-1	4	Bakis-1	4	Ergodic	6	Bakis-1	3	Bakis-1	7	Bakis-2	10	Bakis-1	5
25	“l”	Bakis-1	6	Bakis-1	3	Bakis-1	3	Bakis-1	4	Bakis-1	5	Bakis-1	7	Bakis-1	3	Bakis-1	7	Bakis-2	10	Bakis-1	7
26	“m”	Bakis-1	3	Bakis-1	3	Bakis-1	4	Bakis-1	5	Bakis-1	4	Bakis-2	5	Bakis-1	3	Bakis-1	5	Ergodic	7	Bakis-1	7
27	“n”	Bakis-1	3	Bakis-1	3	Bakis-1	5	Bakis-1	4	Bakis-1	4	Bakis-2	3	Bakis-2	5	Bakis-1	6	Bakis-2	4	Bakis-1	5
28	“ng”	Bakis-1	4	Bakis-1	3	Bakis-1	4	Bakis-1	5	Bakis-1	5	Bakis-1	5	Bakis-1	3	Bakis-1	7	Ergodic	3	Bakis-1	7
29	“p”	Bakis-1	5	Bakis-1	3	Bakis-1	4	Bakis-1	3	Bakis-1	5	Ergodic	3	Bakis-1	3	Ergodic	11	Bakis-1	10	Bakis-1	5
30	“r”	Bakis-1	4	Bakis-1	3	Bakis-1	4	Bakis-1	4	Bakis-1	3	Bakis-2	5	Bakis-1	3	Bakis-1	7	Bakis-2	10	Bakis-1	7
31	“s”	Bakis-1	5	Bakis-1	3	Bakis-1	5	Bakis-1	4	Bakis-1	5	Bakis-2	8	Bakis-1	3	Bakis-1	6	Bakis-2	10	Bakis-1	5
32	“sh”	Bakis-1	3	Bakis-1	3	Bakis-1	5	Bakis-1	3	Bakis-1	5	Bakis-2	10	Bakis-1	3	Bakis-1	6	Ergodic	4	Bakis-1	5
33	“t”	Bakis-1	3	Bakis-1	3	Bakis-1	6	Bakis-1	5	Bakis-1	4	Bakis-1	5	Bakis-1	3	Bakis-1	6	Bakis-2	9	Bakis-1	6
34	“th”	Bakis-1	3	Bakis-1	3	Bakis-1	4	Bakis-1	5	Bakis-1	4	Bakis-1	4	Bakis-1	3	Bakis-1	7	Bakis-2	10	Bakis-1	5
35	“v”	Bakis-1	8	Bakis-1	3	Bakis-1	6	Bakis-1	5	Bakis-1	4	Bakis-2	3	Bakis-1	3	Bakis-2	5	Bakis-2	10	Bakis-1	5
36	“w”	Bakis-1	5	Bakis-1	3	Bakis-1	4	Bakis-1	5	Bakis-1	4	Bakis-1	11	Bakis-2	10	Bakis-2	11	Bakis-2	10	Bakis-1	5
37	“y”	Bakis-1	5	Bakis-1	3	Bakis-1	4	Bakis-1	4	Bakis-1	4	Bakis-2	9	Bakis-1	4	Ergodic	10	Bakis-2	11	Bakis-1	6
38	“z”	Bakis-1	11	Bakis-1	3	Bakis-1	4	Bakis-1	4	Bakis-1	4	Bakis-1	4	Bakis-1	3	Bakis-1	7	Bakis-2	7	Bakis-1	5
39	“zh”	Bakis-2	11	Bakis-1	3	Bakis-1	4	Bakis-1	4	Bakis-1	5	Ergodic	6	Bakis-1	3	Bakis-1	6	Ergodic	7	Bakis-1	6
40	“sil”	Bakis-1	3	Bakis-1	3	Bakis-1	3	Bakis-1	3	Bakis-1	3	Bakis-1	3	Bakis-1	3	Bakis-1	3	Bakis-1	3	Bakis-1	3

No. Bakis-1		37		40		40		40		40		13		37		36		3		40	
No. Bakis-2		2		0		0		0		0		14		3		2		30		0	
No. Ergodic		1		0		0		0		0		13		0		2		7		0	

Average No. States			5.33		3.15		4.45		4.33		4.18		6.88		3.30		6.40		7.95		5.48
Standard Deviation			2.14		0.66		1.04		0.62		0.71		2.55		1.18		1.55		2.77		1.04

No. Gaussian Mixtures		6		6		6		6		7		12		6		6		16		7	

**Table 7:** Recognition accuracies (WAcc) on the testing set with the baseline SD ASR and GA-op ASR systems (source data).

Level of dysarthria	Speaker	Baseline SD ASR		GA-op ASR
		Type	% WAcc	% WAcc
Mild	FB	SD Bakis-1 (3, 6)	81.22	84.72
	MH	SD Bakis-1 (3, 6)	80.35	83.41
	BB	SD Bakis-1 (3, 6)	77.52	79.82
	LL	SD Bakis-1 (3, 6)	74.11	77.23

Moderate	JF	SD Bakis-1 (3, 6)	64.00	66.67
	RL	SD Bakis-1 (3, 6)	51.11	60.00
	RK	SD Ergodic (3, 6)	39.91	47.37

Severe	BK	SD Bakis-1 (3, 6)	36.73	46.46
	BV	SD Bakis-1 (3, 6)	56.82	61.82
	SC	SD Bakis-1 (3, 6)	47.27	54.55

## References

[1] Darley FL, Aronson AE, Brown JR (1969). Differential diagnostic patterns of dysarthria. *Journal of Speech and Hearing Research*.

[2] Darley FL, Aronson AE, Brown JR (1969). Clusters of deviant speech dimensions in the dysarthrias. *Journal of Speech and Hearing Research*.

[3] Kain AB, Hosom J-P, Niu X, van Santen JPH, Fried-Oken M, Staehely J (2007). Improving the intelligibility of dysarthric speech. *Speech Communication*.

[4] Sharma HV (2012). *Acoustic model adaptation for recognition of dysarthric speech [Ph.D. dissertation]*.

[5] Doyle PC, Leeper HA, Kotler AL (1997). Dysarthric speech: a comparison of computerized speech recognition and listener intelligibility. *Journal of Rehabilitation Research and Development*.

[6] Kain A, Niu X, Hosom JP, Miao Q, van Santen JPH Formant re-synthesis of dysarthric speech.

[7] Polur PD, Miller GE (2005). Effect of high-frequency spectral components in computer recognition of dysarthric speech based on a Mel-cepstral stochastic model. *Journal of Rehabilitation Research and Development*.

[8] Hardcastle WJ, Morgan Barry RA, Clark CJ (1985). Articulatory and voicing characteristics of adult dysarthric and verbal dyspraxic speakers: an instrumental study. *British Journal of Disorders of Communication*.

[9] Weismer G, McNeil MR, Rosenbek JC, Aronson AE Articulatory characteristics of Parkinsonian dysarthria: segmental and phrase-level timing, spirantization, and glottal-supraglottal coordination. *The Dysarthrias: Physiology, Acoustics, Perception, Management*.

[10] Kent RD, Rosenbek JC (1983). Acoustic patterns of apraxia of speech. *Journal of Speech and Hearing Research*.

[11] Ackermann H, Hertrich I (1993). Dysarthria in Friedreich’s ataxia: timing of speech segments. *Clinical Linguistics and Phonetics*.

[12] Kim H, Hasegawa-Johnson H, Perlman A Acoustic cues to lexical stress in spastic dysarthria.

[13] Patel R (2002). Prosodic control in severe dysarthria: preserved ability to mark the question-statement contrast. *Journal of Speech, Language, and Hearing Research*.

[14] Kent RD, Kent JF, Duffy JR, Weismer G (1998). The dysarthrias: speech-voice profiles, related dysfunctions, and neuropathology. *Journal of Medical Speech-Language Pathology*.

[15] Ziegler W, Hoole P, Kent RD, Ball MJ (2000). Voice quality measurement. *Neurologic Disease*.

[16] Kent RD, Vorperian HK, Kent JF, Duffy JR (2003). Voice dysfunction in dysarthria: application of the multi-dimensional voice program. *Journal of Communication Disorders*.

[17] Raghavendra P, Rosengren E, Hunnicutt S (2001). An investigation of different degrees of dysarthric speech as input to speaker-adaptive and speaker-dependent recognition systems. *Augmentative and Alternative Communication*.

[18] Caballero Morales SO, Cox SJ (2009). Modelling errors in automatic speech recognition for dysarthric speakers. *EURASIP Journal on Advances in Signal Processing*.

[19] Hamidi F, Baljko M, Livingston N, Miesenberger K, Klaus J, Zagler W, Karshmer A A customizable speech interface for people with dysatric speech.

[20] Rosen K, Yampolsky S (2000). Automatic speech recognition and a review of its functioning with dysarthric speech. *Augmentative and Alternative Communication*.

[21] Ferrier L, Shane H, Ballard H, Carpenter T, Benoit A (1995). Dysarthric speaker’s intelligibility and speech characteristics in relation to computer speech recognition. *Augmentative and Alternative Communication*.

[22] Manasse NJ, Hux K, Rankin-Erickson JL (2000). Speech recognition training for enhancing written language generation by a traumatic brain injury survivor. *Brain Injury*.

[23] Manasse N, Hux K, Rankin-Erickson J, Lauritzen E (2000). Accuracy of three speech recognition systems: case study of dysarthric speech. *Augmentative and Alternative Communication*.

[24] Jayaram G, Abdelhamied K (1995). Experiments in dysarthric speech recognition using artificial neural networks. *Journal of Rehabilitation Research and Development*.

[25] Strik H, Sanders E, Ruiter M, Beijer L Automatic recognition of dutch dysarthric speech: a pilot study.

[26] Matsumasa H, Takiguchi T, Ariki Y, Li I, Nakabayashi T Integration of metamodel and acoustic model for speech recognition.

[27] Hawley MS, Enderby P, Green P (2007). A speech-controlled environmental control system for people with severe dysarthria. *Medical Engineering & Physics*.

[28] Hawley MS, Enderby P, Green P, Cunningham S, Palmer R, Miesenberger K, Klaus J, Zagler WL, Karshmer AI Development of a voice-input voice-output communication aid (VIVOCA) for people with severe dysarthria.

[29] Rabiner LR (1989). A tutorial on hidden Markov models and selected applications in speech recognition. *Proceedings of the IEEE*.

[30] Polur PD, Miller GE (2006). Investigation of an HMM/ANN hybrid structure in pattern recognition application using cepstral analysis of dysarthric (distorted) speech signals. *Medical Engineering & Physics*.

[31] Yakcoub MS, Selouani S-A, O’Shaughnessy D Speech assistive technology to improve the interaction of dysarthric speakers with machines.

[32] Hawley MS, Enderby P, Green P (2013). A voice-input voice-output communication aid for people with severe speech impairment. *IEEE Transactions on Neural Systems and Rehabilitation Engineering*.

[33] Chang H-P Speech input for dysarthric users.

[34] Thomas-Stonell N, Kotler A-L, Leeper HA, Doyle PC (1998). Computerized speech recognition: influence of intelligibility and perceptual consistency on recognition accuracy. *Augmentative and Alternative Communication*.

[35] Hasegawa-Johnson M, Gunderson J, Perlman A, Huang T HMM-based and SVM-based recognition of the speech of talkers with spastic dysarthria.

[36] Seong WK, Park JH, Kim HK, Miesenberger K, Karshmer AI, Penaz P, Zagler WL Dysarthric speech recognition error correction using weighted finite state transducers based on context-dependent pronunciation variation.

[37] Seong WK, Park JH, Kim HK (2012). Multiple pronunciation lexical modeling based on phoneme confusion matrix for dysarthric speech recognition. *Advanced Science and Technology Letters*.

[38] Caballero-Morales SO, Trujillo-Romero F, Batyrshin I, González-Mendoza M (2013). Dynamic estimation of phoneme confusion patterns with a genetic algorithm to improve the performance of metamodels for recognition of disordered speech. *Advances in Computational Intelligence*.

[39] Green P, Carmichael J, Hatzis A, Enderby P, Hawley MS, Parker M Automatic speech recognition with sparse training data for dysarthric speakers.

[40] Frikha M, Hamida AB (2012). A comparative survey of ANN and hybrid HMM/ANN architectures for robust speech recognition. *American Journal of Intelligent Systems*.

[41] Menéndez-Pidal X, Polikoff JB, Peters SM, Leonzio JE, Bunnell HT The nemours database of dysarthric speech.

[42] Chau CW, Kwong S, Man KF, Tang KS (2001). Optimisation of HMM topology and its model parameters by genetic algorithms. *Pattern Recognition*.

[43] Young S, Woodland P (2006). *The HTK Book, (for HTK Version 3.4)*.

[44] Jurafsky D, Martin JH (2009). *Speech and Language Processing*.

[45] Rudzicz F Comparing speaker-dependent and speaker-adaptive acoustic models for recognizing dysarthric speech.

[46] Goldberg DE (1989). *Genetic Algorithms in Search, Optimization and Machine Learning*.

[47] Ferrier LJ, Deller JR, Hsu D (1991). On the use of hidden Markov modelling for recognition of dysarthric speech. *Computer Methods and Programs in Biomedicine*.

[48] Hong QY, Kwong S (2005). A genetic classification method for speaker recognition. *Engineering Applications of Artificial Intelligence*.

[49] Takara T, Iha Y, Nagayama I Selection of the optimal structure of the continuous HMM using the genetic algorithm.

[50] Bakare GA, Venayagamoorthy GK, Aliyu UO Reactive power and voltage control of the Nigerian grid system using micro-genetic algorithm.

[51] Leung KF, Leung FHF, Lam HK, Ling SH (2007). Application of a modified neural fuzzy network and an improved genetic algorithm to speech recognition. *Neural Computing and Applications*.

[52] Kumar B, Dhiman R (2011). Tuning of PID controller for liquid level tank system using intelligent techniques. *International Journal of Computer Science and Technology*.

[53] Kumar R (2010). An experimental analysis of explorative and exploited operators of genetic algorithm for operating system process scheduling problem. *International Journal of Engineering and Technology*.

[54] Nomura T Analysis on linear crossover for real number chromosomes in an infinite population size.

[55] Xiao J, Zou L, Li C Optimization of hidden Markov model by a genetic algorithm for web information extraction.

[56] Vollmer DT, Soule T, Manic M A distance measure comparison to improve crowding in multi-modal optimization problems.

[57] Hosom JP, Kain AB, Mishra T, van Santen JPH, Fried-Oken M, Staehely J Intelligibility of modifications to dysarthric speech.

[58] Seong WK, Park JH, Kim HK (2012). Performance improvement of dysarthric speech recognition using context-dependent pronunciation variation modeling based on Kullback-Leibler distance. *Advanced Science and Technology Letters*.

[59] Bunnel HT, Polikoff JB The nemours database of dysarthric speech: a perceptual analysis.

[60] Gillick L, Cox SJ Some statistical issues in the comparison of speech recognition algorithms.

[61] National Institute of Standards and Technology (NIST) http://www.itl.nist.gov/iad/mig/publications/ASRhistory/index.html.

[62] Noyes JM, Frankish CR (1992). Speech recognition technology for individuals with disabilities. *Augmentative and Alternative Communication*.

